# Supplementary material for: Management of Autism Spectrum Disorder in Italian Units of Child and Adolescent Mental Health: Diagnostic and Referral Pathways
Source: Brain Sci. 2022 Feb 14;12(2):263. doi: 10.3390/brainsci12020263 (PMC8870086; doi:10.3390/brainsci12020263)
Supplement: Supplementary file 1 [file brainsci-12-00263-s001.zip › brainsci-1541475-supplementary - final.pdf]

Table S1

Table S1. Percentage of subjects by ICD-10 ASD classification, CAMH unit and age group (n=634)

CAMH unit	Age group	F84	F84.0	F84.1	F84.5	F84.8	F84.9
**A**	**14-17 yrs**	*0.0*	*47.7*	*6.8*	*36.4*	*0.0*	*9.1*
	**8-11 yrs**	*2.5*	*75.0*	*0.0*	*20.0*	*0.0*	*2.5*
	**1-4 yrs**	*2.3*	*79.1*	*2.3*	*0.0*	*0.0*	*16.3*
**B**	**14-17 yrs**	*43.6*	*33.3*	*2.6*	*7.7*	*2.6*	*10.3*
	**8-11 yrs**	*35.0*	*35.0*	*0.0*	*2.5*	*2.5*	*25.0*
	**1-4 yrs**	*12.2*	*48.8*	*0.0*	*4.9*	*2.4*	*31.7*
**C**	**14-17 yrs**	*0.0*	*46.2*	*0.0*	*7.7*	*0.0*	*46.2*
	**8-11 yrs**	*2.7*	*29.7*	*2.7*	*8.1*	*8.1*	*48.7*
	**1-4 yrs**	*8.8*	*73.5*	*0.0*	*0.0*	*2.9*	*14.7*
**D**	**14-17 yrs**	*0.0*	*38.5*	*2.6*	*15.4*	*7.7*	*35.9*
	**8-11 yrs**	*0.0*	*40.0*	*2.5*	*10.0*	*10.0*	*37.5*
	**1-4 yrs**	*0.0*	*46.8*	*4.3*	*0.0*	*0.0*	*48.9*
**Subtotal (A-D)**	**14-17 yrs**	*12.6*	*40.7*	*3.7*	*19.3*	*3.0*	*20.7*
	**8-11 yrs**	*19.1*	*45.2*	*1.3*	*10.2*	*5.1*	*28.0*
	**1-4 yrs**	*5.5*	*61.2*	*1.8*	*1.2*	*1.2*	*29.1*
**Subtotal (A-D)**	**1-17 yrs**	***12.3***	***49.7***	***2.2***	***9.6***	***3.1***	***26.3***
**E**	**14-17 yrs**	-	-	-	-	-	-
	**8-11 yrs**	*0.0*	*64.3*	*2.4*	*2.4*	*2.4*	*28.6*
	**1-4 yrs**	*1.8*	*78.2*	*1.8*	*0.0*	*0.0*	*18.2*
**F**	**14-17 yrs**	-	-	-	-	-	-
	**8-11 yrs**	*0.0*	*72.5*	*0.0*	*10.0*	*0.0*	*17.5*
	**1-4 yrs**	*5.0*	*92.5*	*2.5*	*0.0*	*0.0*	*0.0*
**Total (A-F)**	**14-17 yrs**	-	-	-	-	-	-
	**8-11 yrs**	*6.7*	*53.1*	*1.3*	*8.8*	*3.8*	*26.4*
	**1-4 yrs**	*4.6*	*69.6*	*1.9*	*0.8*	*0.8*	*22.3*
**Total (A-F)**	**1-11 yrs**	***8.4***	***61.7***	***1.6***	***4.6***	***2.2***	***24.2***

F84: Pervasive developmental disorders; F84.0: Autistic disorder; F84.1: Atypical autism; F84.5: Asperger's syndrome; F84.8: Other pervasive developmental disorders; F84.9: Pervasive developmental disorder, unspecified.

Table S2. Percentage of subjects by mental retardation, CAMH unit and age group (n=566*)

CAMH unit	Age group	None	Mild	Moderate	Severe Profound	Other#
**A**	**14-17 yrs**	*50.0*	*20.5*	*13.6*	*11.4*	*4.6*
	**8-11 yrs**	*37.5*	*45.0*	*5.0*	*2.5*	*10.0*
	**1-4 yrs**	*27.9*	*39.5*	*25.6*	*0.0*	*7.0*
**B**	**14-17 yrs**	*34.2*	*26.3*	*18.4*	*15.8*	*5.3*
	**8-11 yrs**	*31.6*	*15.8*	*23.7*	*23.7*	*5.3*
	**1-4 yrs**	*96.6*	*3.5*	*0.0*	*0.0*	*0.0*
**C**	**14-17 yrs**	*10.0*	*30.0*	*20.0*	*40.0*	*0.0*
	**8-11 yrs**	*33.3*	*13.3*	*33.3*	*6.7*	*13.3*
	**1-4 yrs**	*0.0*	*38.9*	*11.1*	*0.0*	*50.0*
**D**	**14-17 yrs**	*56.4*	*18.0*	*20.5*	*5.1*	*0.0*
	**8-11 yrs**	*72.5*	*10.0*	*5.0*	*2.5*	*10.0*
	**1-4 yrs**	*89.4*	*0.0*	*0.0*	*0.0*	*10.6*
**Subtotal (A-D)**	**14-17 yrs**	*44.3*	*22.1*	*17.6*	*13.0*	*3.1*
	**8-11 yrs**	*45.9*	*22.6*	*13.5*	*9.0*	*9.0*
	**1-4 yrs**	*59.9*	*18.2*	*9.5*	*0.0*	*12.4*
**Subtotal (A-D)**	**1-17 yrs**	***50.1***	***20.9***	***13.5***	***7.2***	***8.2***
**E**	**14-17 yrs**	-	-	-	-	-
	**8-11 yrs**	*71.8*	*12.8*	*10.3*	*5.1*	*0.0*
	**1-4 yrs**	*65.2*	*15.2*	*8.7*	*10.9*	*0.0*
**F**	**14-17 yrs**	-	-	-	-	-
	**8-11 yrs**	*85.0*	*10.0*	*0.0*	*5.0*	*0.0*
	**1-4 yrs**	*15.0*	*67.5*	*17.5*	*0.0*	*0.0*
**Total (A-F)**	**14-17 yrs**	-	-	-	-	-
	**8-11 yrs**	*58.0*	*18.4*	*10.4*	*7.5*	*5.7*
	**1-4 yrs**	*52.9*	*26.5*	*10.8*	*2.2*	*7.6*
**Total (A-F)**	**1-11 yrs**	***55.4***	***22.5***	***10.6***	***4.8***	***6.7***

*Information is not available for 68 (10.7%) children and adolescents (14-17 yrs: n=4; 8-11 yrs: n=27; 1-4 yrs: n=37). #Other and Unspecified intellectual disabilities

Table S3

Table S3. Percentage of subjects by challenging behaviours and Mental retardation, CAMH unit and age group (n=485*)

	**Challenging behaviours:**	**Yes**	**Yes**	**No**	**No**
	**Mental retardation:**	**Yes**	**No**	**Yes**	**No**
**CAMH unit**	**Age group**	%	%	%	%
**A**	**14-17 yrs**	*16.3*	*7.0*	*34.9*	*41.9*
	**8-11 yrs**	*25.6*	*5.1*	*35.9*	*33.3*
	**1-4 yrs**	*12.2*	*2.4*	*61.0*	*24.4*
**B**	**14-17 yrs**	*38.9*	*13.9*	*27.8*	*19.4*
	**8-11 yrs**	*45.5*	*15.2*	*21.2*	*18.2*
	**1-4 yrs**	*3.7*	*11.1*	*0.0*	*85.2*
**C**	**14-17 yrs**	*62.5*	*0.0*	*25.0*	*12.5*
	**8-11 yrs**	*50.0*	*0.0*	*8.3*	*41.7*
	**1-4 yrs**	*100.0*	*0.0*	*0.0*	*0.0*
**D**	**14-17 yrs**	*31.8*	*13.6*	*27.3*	*27.3*
	**8-11 yrs**	*16.2*	*21.6*	*13.5*	*48.6*
	**1-4 yrs**	*4.8*	*57.1*	*9.5*	*28.6*
**Subtotal (A-D)**	**14-17 yrs**	*30.3*	*10.1*	*30.3*	*29.4*
	**8-11 yrs**	*30.6*	*12.4*	*22.3*	*34.7*
	**1-4 yrs**	*16.3*	*16.3*	*27.6*	*39.8*
**Subtotal (A-D)**	**1-17 yrs**	***26.2***	***12.8***	***26.5***	***34.5***
**E**	**14-17 yrs**	-	-	-	-
	**8-11 yrs**	*9.1*	*12.1*	*18.2*	*60.6*
	**1-4 yrs**	*13.3*	*4.4*	*20.0*	*62.2*
**F**	**14-17 yrs**	-	-	-	-
	**8-11 yrs**	*10.3*	*46.2*	*5.1*	*38.5*
	**1-4 yrs**	*80.0*	*10.0*	*5.0*	*5.0*
**Total (A-F)**	**14-17 yrs**	-	-	-	-
	**8-11 yrs**	*22.8*	*19.2*	*18.1*	*39.9*
	**1-4 yrs**	*29.5*	*12.0*	*20.8*	*37.7*
**Total (A-F)**	**1-11 yrs**	***26.1***	***15.7***	***19.4***	***38.8***

*Information on challenging behaviours and/or Mental retardation is not available for 149 (23.5%) children and adolescents (14-17 yrs: n=26; 8-11 yrs: n=46; 1-4 yrs: n=77).

Table S4

Table S4. Significance levels of the comparisons among age groups in each CAMH unit or groups of units

	CAMH units	**Age groups:**		**14-17 vs**	**8-11 vs**	**1-4 yrs**	**Age groups:**		**8-11 vs**	**1-4 yrs**			
**Variable**	**Test**	**A**	**B**	**C**	**D**	**A-D**	**A**	**B**	**D**	**C**	**E**	**F**	**A-F**
Diagnostic category	Fisher p	**<0.001**	**0.031**	**0.003**	**0.044**	**<0.001**	**0.001**	0.139	**0.021**	**<0.001**	0.303	**<0.001**	**<0.001**
Mental retardation	Fisher p	**0.008**	**<0.001**	**<0.001**	**<0.001**	**<0.001**	***0.075***	**<0.001**	**0.035**	**0.003**	0.789	**<0.001**	**0.030**
Challenging behaviour & Mental retardation	Fisher p	0.175	**<0.001**	**0.041**	**0.012**	0.114	0.125	**<0.001**	**0.059**	**0.023**	0.664	**<0.001**	0.163
Mental health conditions	Fisher p	**<0.001**	**0.008**	0.173	0.227	**<0.001**	**<0.001**	**0.007**	***0.107***	***0.096***	0.753	**<0.001**	**0.013**
Physical health conditions	Fisher p	0.629	**0.023**	0.130	***0.073***	0.180	0.573	**0.011**	1.000	***0.060***	0.456	0.237	0.495
Referrer	Fisher p	**<0.001**	**<0.001**	**0.032**	**0.004**	**<0.001**	**0.001**	**<0.001**	***0.088***	**0.033**	0.420	0.223	**<0.001**
Neurodevelopmental concern	Fisher p	**0.009**	**<0.001**	**0.047**	***0.051***	**0.006**	0.261	**<0.001**	***0.084***	**0.032**	0.691	**0.035**	**<0.001**
Age at diagnosis	KW p	**<0.001**	**<0.001**	**<0.001**	**0.009**	**<0.001**	**<0.001**	**0.004**	**0.003**	**<0.001**	**<0.001**	**<0.001**	**<0.001**
Team quality	Fisher p	**0.001**	**0.001**	**0.004**		**0.002**	**0.011**	**0.008**		0.220	**0.034**	**<0.001**	0.136
Standardized tools													
ASD diagnosis	Fisher p	**<0.001**	**0.005**	**0.001**	**<0.001**	**<0.001**	0.212	**0.002**	**0.023**	**<0.001**	0.433		**<0.001**
Cognitive	Fisher p	0.357	**<0.001**	**0.009**	**<0.001**	**<0.001**	0.248	**0.001**	**<0.001**	**0.006**	**0.009**	**<0.001**	**0.001**
Adaptive skills	Fisher p	0.202	**<0.001**	1.000	**<0.001**	***0.068***	1.000	**0.002**	0.001	1.000	0.256	0.241	0.791
Psychoeduc. profile	Fisher p	***0.066***	**<0.001**	1.000		**<0.001**		**<0.001**		1.000	0.476	**0.002**	**0.005**
Laboratory tests													
Aux	Fisher p	0.502	**<0.001**	0.569	**0.046**	**0.019**	0.710	**<0.001**	0.066	0.420		0.495	0.118
Eye	Fisher p	1.000	**0.009**	0.164	0.668	***0.074***	1.000	**0.020**	1.000	0.188	1.000	0.401	**0.002**
Aud	Fisher p	***0.068***	0.137	0.942	0.276	0.821	***0.066***	***0.066***	0.284	0.783	1.000	0.262	0.139
Gen	Fisher p	**0.020**	**<0.001**	0.592	0.210	0.278	0.139	**0.001**	0.141	0.602	0.638	**0.002**	0.170
Card	Fisher p	0.556	0.475	**0.002**	0.160	**0.003**	0.412	0.433	0.099	0.237	1.000	1.000	**0.040**
EEG	Fisher p	0.426	1.000	0.586	**0.036**	***0.055***	0.290	1.000	0.024	0.386	0.311	0.161	**0.007**
EP	Fisher p	0.612	***0.090***	0.887	1.000	0.659	0.476	***0.100***		0.707	**0.019**	**<0.001**	**<0.001**
MRI	Fisher p	***0.066***	0.734	0.256	0.134	***0.090***	**0.040**	0.589	0.063	0.181	1.000	0.263	**0.049**
Food	Fisher p	0.612	0.552	1.000	0.612	0.194	0.476	0.446	1.000	1.000	1.000	0.423	0.266

Met	Fisher p	1.000	0.137	1.000	**<0.001**	***0.059***	1.000	***0.066***	<0.001	1.000		1.000	0.328
Ant	Fisher p	0.187	0.776	0.198	0.332	0.608	0.223	0.504		0.228			0.445
End	Fisher p	1.000	***0.066***	1.000	***0.058***	***0.056***	1.000	0.165	0.622	1.000		***0.051***	**0.001**
Written report	Fisher p	0.539		0.495		0.264	0.494			0.429	**0.001**		**0.002**
Referral to first visit	KW p		0.500	0.141	0.666	0.925		0.250	0.391	0.238	0.511	**0.010**	***0.063***
Referral to first visit (<=90 days)	Fisher p		0.494	0.732	0.741	1.000			0.499	1.000	0.352	**0.002**	**0.004**
First visit to diagnosis	KW p	0.276	**0.015**	0.292	**0.024**	**0.002**	***0.077***	0.212	0.002	0.856	0.790	0.736	**0.016**
First visit to diagnosis (<=90 days)	Fisher p	0.505	**0.002**	0.426	0.148	***0.063***	0.297	***0.0920***	0.073	0.460	1.000	0.474	***0.100***
Diagnosis to intervention	KW p	0.339	**<0.001**	0.526	**0.018**	**<0.001**	0.156	**0.001**	0.006	0.753	0.131	0.655	**<0.001**
Diagnosis to intervention (<=180 days)	Fisher p	0.405	**<0.001**	0.673	0.213	**<0.001**	0.202	**<0.001**	0.182	0.458	0.147	0.777	**<0.001**

Significance levels: bold p<=0.05; bold+italic 0.05<p<=0.10

Empy cells refer to comparisons where all subjects were classified in one single level of the categorical variable

Abbreviations. Aux: Auxological parameters; Eye: Eye examination; Aud: Audiometric investigation; Gen: Genetic/karyotype investigations; Card: Cardiological examination; EEG: Electroencephalography; EP: Evoked potentials; MRI: Magnetic resonance imaging; Food: Food intolerance; Met: Metabolic investigation; Ant: Quantitative dosage of antibodies; End: Endocrine investigations; Fisher = Fisher's exact probability test; KW = Kruskal-Wallis test.

Table S5

Table S5. Frequency of subjects by laboratory tests characterization, CAMH unit and age group

CAMH unit	Age Group	Aux		Eye		Aud		Gen		Card		EEG		EP		MRI		Food		Met		Ant		End	
		n	%	n	%	n	%	n	%	n	%	n	%	n	%	n	%	n	%	n	%	n	%	n	%
**A**	**14-17 yrs**	1	*4.6*	2	*9.1*	5	*22.7*	11	*50.0*	3	*13.6*	14	*63.6*	0	*0.0*	11	*50.0*	1	*0.0*	2	*9.1*	0	*0.0*	0	*0.0*
	**8-11 yrs**	3	*10.0*	3	*10.0*	7	*23.3*	20	*66.7*	4	*13.3*	22	*73.3*	1	*3.3*	22	*73.3*	1	*3.3*	4	*13.3*	2	*6.7*	1	*3.3*
	**1-4 yrs**	5	*15.2*	4	*12.1*	16	*48.5*	28	*84.9*	2	*6.1*	19	*57.6*	0	*0.0*	15	*45.5*	0	*0.0*	4	*12.1*	0	*0.0*	1	*3.0*
**B**	**14-17 yrs**	16	*53.3*	15	*50.0*	15	*50.0*	15	*50.0*	20	*66.7*	26	*86.7*	7	*23.3*	12	*40.0*	3	*10.0*	15	*50.0*	1	*3.3*	17	*56.7*
	**8-11 yrs**	12	*36.4*	14	*42.4*	18	*54.6*	15	*45.5*	17	*51.5*	28	*84.9*	9	*27.3*	13	*39.4*	6	*18.2*	15	*45.5*	2	*6.1*	15	*45.5*
	**1-4 yrs**	21	*87.5*	3	*12.5*	7	*29.2*	1	*4.2*	15	*62.5*	21	*87.5*	12	*50.0*	12	*50.0*	2	*8.3*	17	*70.8*	0	*0.0*	6	*25.0*
**C**	**14-17 yrs**	5	*50.0*	2	*20.0*	6	*60.0*	3	*30.0*	4	*40.0*	7	*70.0*	1	*10.0*	3	*30.0*	1	*10.0*	1	*10.0*	1	*10.0*	1	*10.0*
	**8-11 yrs**	11	*37.9*	15	*51.7*	19	*65.5*	14	*48.3*	3	*10.3*	22	*75.9*	5	*17.2*	8	*27.6*	5	*17.2*	3	*10.3*	0	*0.0*	3	*10.3*
	**1-4 yrs**	14	*51.9*	9	*33.3*	16	*59.3*	11	*40.7*	0	*0.0*	17	*63.0*	3	*11.1*	3	*11.1*	4	*14.8*	3	*11.1*	2	*7.4*	2	*7.4*
**D**	**14-17 yrs**	18	*94.7*	4	*21.1*	12	*63.2*	6	*31.6*	7	*36.8*	14	*73.7*	1	*5.3*	11	*57.9*	0	*0.0*	9	*47.4*	2	*10.5*	0	*0.0*
	**8-11 yrs**	13	*72.2*	2	*11.1*	10	*55.6*	10	*55.6*	7	*38.9*	14	*77.8*	0	*0.0*	14	*77.8*	1	*5.6*	0	*0.0*	0	*0.0*	4	*22.2*
	**1-4 yrs**	12	*100.0*	1	*8.3*	4	*33.3*	3	*25.0*	1	*8.3*	4	*33.3*	0	*0.0*	5	*41.7*	0	*0.0*	7	*58.3*	0	*0.0*	1	*8.3*
**Subtotal (A-D)**	**14-17 yrs**	40	*49.4*	23	*28.4*	38	*46.9*	35	*43.2*	34	*42.0*	61	*75.3*	9	*11.1*	37	*45.7*	5	*6.2*	27	*33.3*	4	*4.9*	18	*22.2*
	**8-11 yrs**	39	*35.5*	34	*30.9*	54	*49.1*	59	*53.6*	31	*28.2*	86	*78.2*	15	*13.6*	57	*51.8*	13	*11.8*	22	*20.0*	4	*3.6*	23	*20.9*
	**1-4 yrs**	52	*54.2*	17	*17.7*	43	*44.8*	43	*44.8*	18	*18.8*	61	*63.5*	15	*15.6*	35	*36.5*	6	*6.3*	31	*32.3*	2	*2.1*	10	*10.4*
**E**	**14-17 yrs**	-	-	-	-	-	-	-	-	-	-	-	-	-	-	-	-	-	-	-	-	-	-	-	-
	**8-11 yrs**	0	*0.0*	0	*0.0*	5	*38.5*	11	*84.6*	0	*0.0*	0	*0.0*	1	*7.7*	2	*15.4*	0	*0.0*	0	*0.0*	0	*0.0*	0	*0.0*
	**1-4 yrs**	0	*0.0*	1	*2.6*	13	*34.2*	34	*89.5*	1	*2.6*	5	*13.2*	17	*44.7*	7	*18.4*	1	*2.6*	0	*0.0*	0	*0.0*	0	*0.0*
**F**	**14-17 yrs**	-	-	-	-	-	-	-	-	-	-	-	-	-	-	-	-	-	-	-	-	-	-	-	-
	**8-11 yrs**	5	*16.7*	19	*63.3*	19	*63.3*	10	*33.3*	11	*36.7*	22	*73.3*	5	*16.7*	10	*33.3*	0	*0.0*	6	*20.0*	0	*0.0*	10	*33.3*
	**1-4 yrs**	6	*27.3*	11	*50.0*	10	*45.5*	17	*77.3*	8	*36.4*	20	*90.9*	16	*72.7*	11	*50.0*	1	*4.6*	5	*22.7*	0	*0.0*	2	*9.1*
**Total (A-F)**	**8-11 yrs**	44	*28.8*	53	*34.6*	78	*51.0*	80	*52.3*	42	*27.5*	108	*70.6*	21	*13.7*	69	*45.1*	13	*8.5*	28	*18.3*	4	*2.6*	33	*21.6*
	**1-4 yrs**	58	*37.2*	29	*18.6*	66	*42.3*	94	*60.3*	27	*17.3*	86	*55.1*	48	*30.8*	53	*34.0*	8	*5.1*	36	*23.1*	2	*1.3*	12	*7.7*
**Total (A-D, F)**	**8-11 yrs**	44	*31.4*	53	*37.9*	73	*52.1*	69	*49.3*	42	*30.0*	108	*77.1*	20	*14.3*	67	*47.9*	13	*9.3*	28	*20.0*	4	*2.9*	33	*23.6*
	**1-4 yrs**	58	*49.2*	28	*23.7*	53	*44.9*	60	*50.9*	26	*22.0*	81	*68.6*	31	*26.3*	46	*39.0*	7	*5.9*	36	*30.5*	2	*1.7*	12	*10.2*

Note. Since participants could list more than one Lab test (multiple answers could be selected), the sum of individual percentages exceeds 100%.

Abbreviations. Aux: Auxological parameters; Eye: Eye examination; Aud: Audiometric investigation; Gen: Genetic/karyotype investigations; Card: Cardiological examination; EEG: Electroencephalography; EP: Evoked potentials; MRI: Magnetic resonance imaging; Food: Food intolerance; Met: Metabolic investigation; Ant: Quantitative dosage of antibodies; End: Endocrine investigations

Table S6

Table S6. Frequency of subjects receiving written assessment report, by CAMH unit and age group

CAMH unit	Age group	Subjects n	Yes n	%
**A**	**14-17 yrs**	34	33	*97.1*
	**8-11 yrs**	39	38	*97.4*
	**1-4 yrs**	40	40	*100.0*
**B**	**14-17 yrs**	33	33	*100.0*
	**8-11 yrs**	31	31	*100.0*
	**1-4 yrs**	40	40	*100.0*
**C**	**14-17 yrs**	6	5	*83.3*
	**8-11 yrs**	6	5	*83.3*
	**1-4 yrs**	8	8	*100.0*
**D**	**14-17 yrs**	39	39	*100.0*
	**8-11 yrs**	40	40	*100.0*
	**1-4 yrs**	47	47	*100.0*
**Subtotal (A-D)**	**14-17 yrs**	112	110	*98.2*
	**8-11 yrs**	116	114	*98.3*
	**1-4 yrs**	135	135	*100.0*
**E**	**14-17 yrs**	-	-	-
	**8-11 yrs**	31	21	*67.7*
	**1-4 yrs**	51	49	*96.1*
**F**	**14-17 yrs**	-	-	-
	**8-11 yrs**	40	40	*100.0*
	**1-4 yrs**	40	40	*100.0*
**Total (A-F)**	**8-11 yrs**	187	175	*93.6*
	**1-4 yrs**	226	224	*99.1*

Table S7. Frequency of subjects with waiting time characterising diagnosis process under the cut-off (referral to first visit <=90 days, first visit to diagnosis <=90 days, diagnosis to intervention <=180 days), by CAMH unit and age group

**CAMH units**	**Age group**	***referral to first visit (<= 90 days)***		***first visit to diagnosis (<= 90 days)***		***diagnosis to intervention (<= 180 days)***	
		n/TOT	%	n/TOT	%	n/TOT	%
**A**	**14-17 yrs**	-	-	13/35	*37.1*	8/17	*47.1*
	**8-11 yrs**	-	-	9/32	*28.1*	6/17	*35.3*
	**1-4 yrs**	-	-	13/31	*41.9*	11/19	*57.9*
**B**	**14-17 yrs**	18/19	*94.7*	17/30	*56.7*	4/19	*21.1*
	**8-11 yrs**	19/19	*100.0*	24/31	*77.4*	12/33	*36.4*
	**1-4 yrs**	39/39	*100.0*	37/40	*92.5*	37/38	*97.4*
**C**	**14-17 yrs**	10/10	*100.0*	3/12	*25.0*	5/12	*41.7*
	**8-11 yrs**	28/31	*90.3*	16/34	*47.1*	16/35	*45.7*
	**1-4 yrs**	28/32	*90.3*	12/33	*36.4*	11/31	*35.5*
**D**	**14-17 yrs**	29/32	*90.6*	14/34	*41.2*	10/28	*35.7*
	**8-11 yrs**	31/34	*91.2*	10/40	*25.0*	13/36	*36.1*
	**1-4 yrs**	35/41	*85.4*	21/47	*44.7*	25/47	*53.2*
**Subtotal (A-D)**	**14-17 yrs**	57/61	*93.4*	47/111	*42.3*	27/76	*35.5*
	**8-11 yrs**	78/102	*92.9*	59/137	*43.1*	47/121	*38.8*
	**1-4 yrs**	102/111	*91.9*	83/151	*55.0*	84/135	*62.2*
**E**	**14-17 yrs**	-	-	-	-	-	-
	**8-11 yrs**	24/25	*96.0*	19/24	*79.2*	2/6	*33.3*
	**1-4 yrs**	46/46	*100.0*	37/46	*80.4*	16/22	*72.7*
**F**	**14-17 yrs**	-	-	-	-	-	-
	**8-11 yrs**	20/40	*50.0*	29/40	*72.5*	7/17	*41.2*
	**1-4 yrs**	34/40	*85.0*	25/40	*62.5*	17/37	*46.0*
**Total (A-F)**	**8-11 yrs**	122/149	*81.9*	107/201	*53.2*	56/144	*38.9*
	**1-4 yrs**	187/197	*92.4*	145/237	*61.2*	117/194	*60.3*
